# Device Closure of Hemodynamically Significant Patent Ductus Arteriosus in Premature Infants

**DOI:** 10.1016/j.jacadv.2024.101211

**Published:** 2024-08-24

**Authors:** Alban-Elouen Baruteau, Mathilde Méot, Nadir Benbrik, Céline Grunenwald, Naychi Lwin, Juliana Patkai, Jean-Christophe Rozé, Damien Bonnet, Sophie Malekzadeh-Milani

**Affiliations:** a Department of Pediatric Cardiology and Pediatric Cardiac Surgery, Nantes Université, CHU Nantes, FHU PreciCare, Nantes, France; b Nantes Université, CHU Nantes, INSERM, CIC FEA 1413, Nantes, France; c Nantes Université, CHU Nantes, CNRS, INSERM, l’institut du thorax, Nantes, France; d Nantes Université, INRAE, UMR 1280, PhAN, Nantes, France; e M3C-Necker, Department of Pediatric and Congenital Cardiology, Hôpital Necker-Enfants Malades, Assistance Publique-Hôpitaux de Paris (AP-HP), Paris, France; f Department of Neonatal Medicine, Cochin-Port Royal Hospital, FHU PREMA, APHP, Paris, France; g University Paris Cité, Paris, France

**Keywords:** echocardiography, extremely low birth weight infants, patent ductus arteriosus, premature infant, outcomes

## Abstract

The patent ductus arteriosus is a very common condition in preterm infants, and a hemodynamically significant patent ductus arteriosus increases morbidity and mortality in these vulnerable patients. However, despite numerous randomized controlled trials, there is no consensus regarding management. Medical therapy is typically offered as first-line treatment, although it yields limited success and carries the potential for severe adverse events. In recent years, there has been rapid development in transcatheter patent ductus arteriosus closure primary with the use of the Amplatzer Piccolo Occluder, and this has gained widespread acceptance as a safe and effective alternative to surgical ligation in extremely low-birth-weight infants weighing over 700 g. This article aims to provide an appraisal of the patient selection process, a step-by-step procedural guide, and a comprehensive review of the outcomes associated with this approach.

## Highlights

• Surgical ligation has traditionally been the standard of care for hemodynamically significant patent ductus arteriosus in extremely low-birth-weight premature infants. However, transcatheter closure is emerging as a safe and effective alternative in experienced centers.
• Optimal patient selection and management requires multidisciplinary involvement.
• The procedure relies on close collaboration between the interventionalist and the sonographer performing the transthoracic echocardiogram, as it is essential in confirming the indication for closure, choosing the right device and guiding appropriate device placement.
• Modified techniques including bedside procedures and fluoroscopy-free approaches, combined with the increasing expertise of interventionalists and echocardiographers, will further enhance the safety and broaden the application of transcatheter patent ductus arteriosus closure in these extremely vulnerable patients.

**Figure 1:**
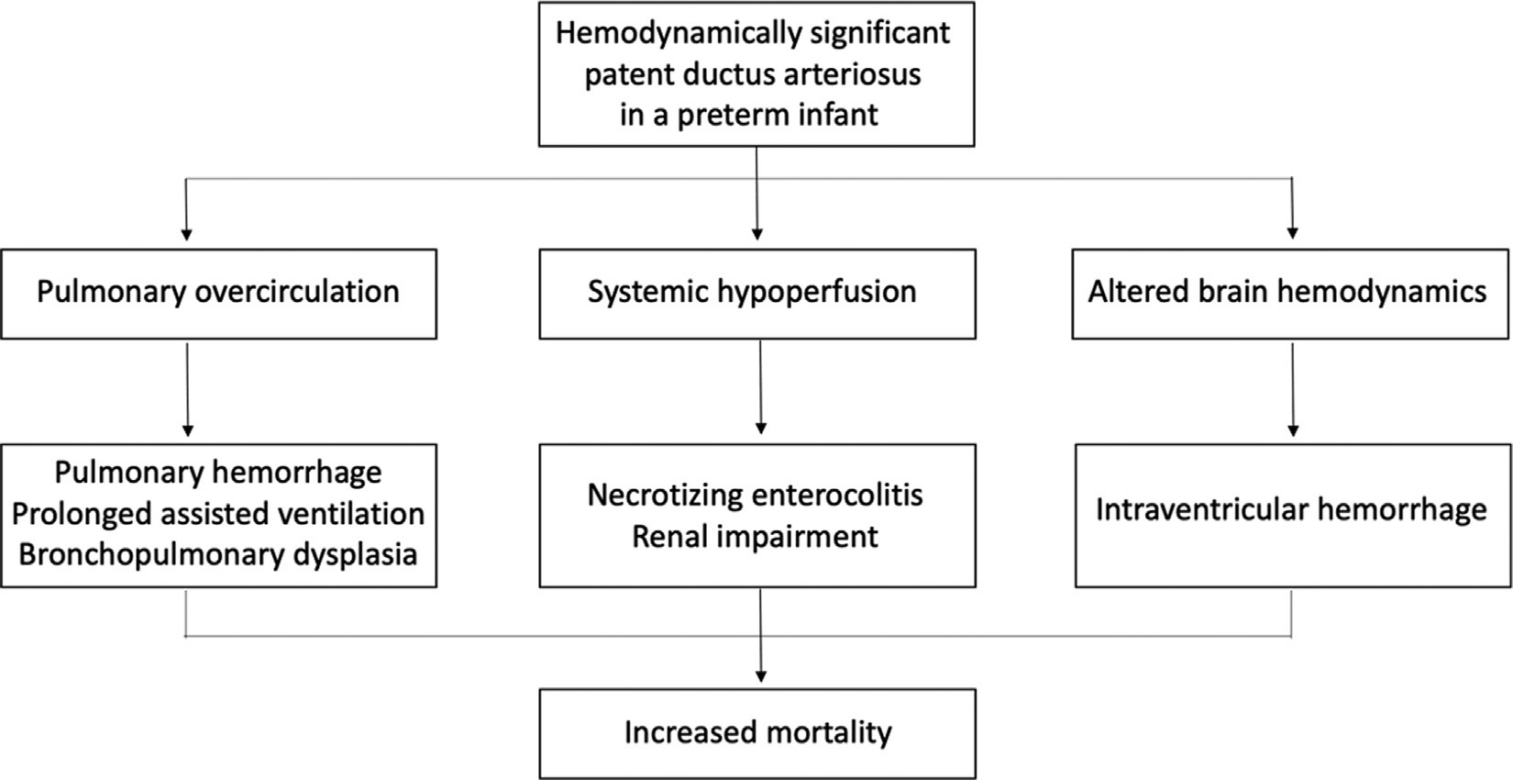
**Pathophysiology and Clinical Consequences of a Hemodynamically Significant Patent DuctusArteriosus in an Extremely Low-Birth-Weight Infant**

In preterm infants, a hemodynamically significant patent ductus arteriosus (hsPDA) increases the risk of chronic respiratory disease, prolonged assisted ventilation, pulmonary hemorrhage, bronchopulmonary dysplasia, intraventricular hemorrhage, renal impairment, necrotizing enterocolitis (NEC), and death^1^ (Central Illustration, Figure 1). Active treatment for closing a PDA remains controversial, as conservative management in premature infants demonstrate comparable outcomes to early ibuprofen administration in regards to NEC, bronchopulmonary dysplasia, or death at 36 weeks’ postmenstrual age.^2^^,^^3^ Furthermore, a PDA will close spontaneously by day 7 of life in 77% of preterm infants born under 30 weeks gestation and in 41% of those born under 28 weeks gestation. The rate of spontaneous closure of a PDA has been reported to be inversely proportional to the gestational age.^1^ Sung et al. demonstrated that in 167 infants born between 23 to 28 weeks gestation, 95% had spontaneous PDA closure at hospital discharge,^4^ supporting a conservative approach to PDA management. However, clinical observational studies consistently show a strong association between an hsPDA and neonatal morbidity, supporting a more active PDA treatment approach. Therefore, PDA closure may be indicated in any preterm infant with a hsPDA. Cyclo-oxygenase inhibitors and/or paracetamol are typically used as initial medical treatment exhibiting a moderate success rate of 60 to 70%. However, their use carries the risk of major adverse events (MAE), such as bleeding, NEC, renal impairment, and developmental delay.^5^ Surgical PDA ligation has traditionally been the sole nonpharmacological option; however, transcatheter PDA closure has emerged as a safe and effective alternative for infants who have not responded to medical management nor have contraindications to it. The Amplatzer Piccolo Occluder (APO, Abbott Structural Heart) received Food and Drug Administration (FDA) and Conformité Européenne-mark approval in 2019 for PDA closure in infants weighing more than 700 g and older than 3 days of life, with an excellent success rate and a low incidence of periprocedural complications when performed in experienced teams.^6^ It has now become the preferred therapeutic option over surgical ligation in many expert centers worldwide. This article aims to provide an overview of the case and device selection process, a step-by-step description of the procedure, and a contemporary review of its outcomes.

**Central Illustration:**
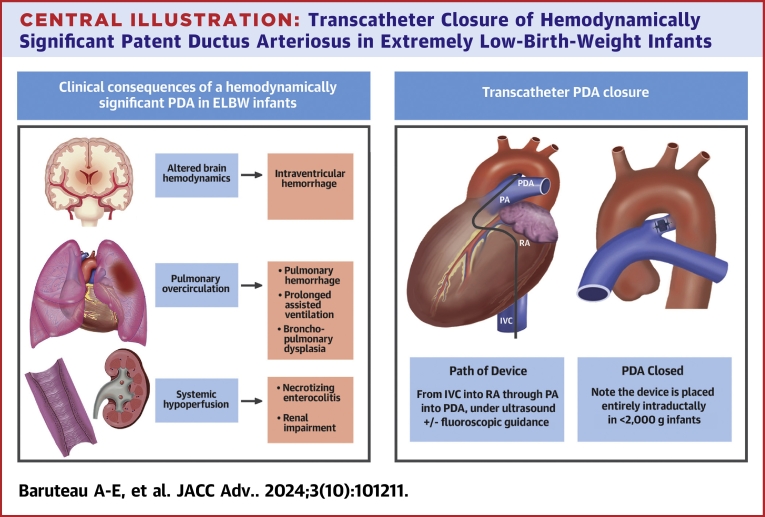
**Transcatheter Closure of Hemodynamically Significant Patent Ductus Arteriosus in Extremely Low-Birth-Weight Infants** ELBW = extremely low-birth-weight; IVC = inferior vena cava; PA = pulmonary artery; PDA = patent ductus arteriosus; RA = right atrium.

## Definitions: epidemiology

A PDA is defined as a failure of the ductus arteriosus to close within 72 hours after birth. It is a common condition in very low-birth-weight (VLBW, birth weight <1,500 g) and extremely low-birth-weight (ELBW) (birth weight <1,000 g) infants, with a higher incidence in lower gestational age. It is reported in around 50% of the infants born <28 weeks gestation and/or at a birth weight <1,000 g.^7^

A comprehensive definition of a hsPDA would encompass the magnitude of the ductal shunt and the hemodynamic impact, integrating various parameters such as PDA size, shunt volume, cardiovascular load, and end-organ perfusion. This definition would offer guidance on which patients requirement treatment and the optimal timing for intervention. Since the first staging system proposed by McNamara et al.^8^ in 2007, several PDA scoring systems have been developed for risk stratification, to help clinicians identify higher-risk infants who may benefit from PDA closure.^8^^,^^9^ The definition of a hsPDA is based on a staging system that incorporates both clinical and echocardiographic criteria. Clinical indicators include the need for respiratory support, mechanical ventilation or high-frequency ventilation, feeding intolerance or abdominal distension resembling NEC, acute renal failure, hemodynamic instability, or metabolic acidosis. Echocardiographic criteria include a transductal diameter >1.5 mm, left heart volume loading, decreased, absent, or reversed diastolic flow in the superior mesenteric artery, middle cerebral artery, or renal artery and unrestrictive pulsatile transductal flow.^1^^,^^5^^,^^8^^,^^9^ These criteria are detailed in Table 1. This purpose of this staging system for PDA categorization is to facilitate triage and case prioritization, particularly for the most critically ill infants. However, there is currently no standardized consensus in defining a hemodynamically significant PDA in a preterm infant.

**Table 1:** Clinical and Echocardiographic Criteria for Determining the Magnitude of a Hemodynamically Significant PDA

Clinical Criteria	
C1	Asymptomatic
C2	Mild
	Oxygenation difficulty (OI <6)
	Occasional (<6) episodes of oxygen desaturation, bradycardia, or apnea
	Need for respiratory support (nCPAP) or mechanical ventilation (MAP <8)
	Feeding intolerance (>20% gastric aspirates)
	Radiologic evidence of increased pulmonary vasoreactivity
C3	Moderate
	Oxygenation difficulty (OI 7-14)
	Frequent (hourly) episodes of oxygen desaturation, bradycardia, or apnea
	Increasing ventilation requirements (MAP 9-12)
	Inability to feed due to marked abdominal distension or emesis
	Oliguria with mild elevation in plasma creatinine
	Systemic hypotension (low mean or diastolic BP) requiring a single cardiotropic agent
	Radiological evidence of cardiomegaly or pulmonary edema
	Mild metabolic acidosis (pH 7.1-7.25 and/or base deficit −7 to −12.0)
C4	Severe
	Oxygenation difficulty (OI >15)
	High ventilation requirements (MAP >12) or need for high-frequency modes of ventilation
	Profound or recurrent pulmonary hemorrhage
	NEC-like abdominal distension with tenderness or erythema
	Acute renal failure
	Hemodynamic instability requiring >1 cardiotropic agent
	Moderate to severe metabolic acidosis (pH <7.1 or base deficit >−12.0)

Echocardiographic Criteria	
E1	No evidence of ductal flow on 2-dimensional or Doppler interrogation
E2	Small nonsignificant ductus arteriosus
	Transductal diameter <1.5 mm
	Restrictive continuous transductal flow (DA Vmax >2.0 m/s)
	No signs of left heart volume loading (eg, LA:Ao ratio >1.5:1)
	No signs of left heart pressure loading (eg, E/A ratio >1.0)
	Normal end-organ diastolic flow in superior mesenteric artery, middle cerebral artery, or renal artery
E3	Moderate hemodynamically significant PDA
	Transductal diameter 1.5-3.0 mm
	Unrestrictive pulsatile transductal flow (DA Vmax <2.0 m/s)
	Mild to moderate left heart volume loading (eg, LA:Ao ratio 1.5-2:1)
	Mild to moderate left heart pressure loading (eg, E/A ratio >1.0)
	Decreased or absent end-organ diastolic flow in superior mesenteric artery, middle cerebral artery, or renal artery
E4	Large hemodynamically significant PDA
	Transductal diameter >3.0 mm
	Unrestrictive pulsatile transductal flow
	Severe left heart volume loading (eg, LA:Ao ratio >2:1)
	Severe left heart pressure loading (eg, E/A ratio >1.5)
	Reversal of end-diastolic flow in superior mesenteric artery, middle cerebral artery, or renal artery

Patients should be assigned both a clinical (C1 to C4) and an echocardiographic (E1 to E4) stage.

Adapted From McNamara et al.^8^

BP = blood pressure; DA Vmax = ductus arteriosus peak velocity; E/A = early passive to late atrial contractile phase of transmitral filling ratio; LA:Ao = left atrium to aortic; MAP = mean airway pressure; nCPAP = nasal continuous positive airway pressure; NEC = necrotizing enterocolitis; OI = oxygenation index; PDA = patent ductus arteriosus.

## Case selection, indications, and contra indications

PDA closure may be indicated in any symptomatic preterm infant with a hemodynamically significant PDA. There are a few contraindications to transcatheter PDA closure in ELBW infants which are summarized in Table 2. High-frequency ventilation does not compromise the safe and efficient placement of devices.^10^ Renal failure, cerebral hemorrhage, or enterocolitis does not preclude the procedure, providing there is no sepsis. The APO device is not recommended for use in the very large (ie, >4 mm minimal ductal diameter) PDA, which may necessitate considering exemption or even off-label use of alternative devices, such as the Amplatzer Vascular Plug-II (AVP-II, Abbott).

**Table 2:** Indications and Contraindications for Transcatheter PDA Closure in Extremely Low-Birth-Weight Infants

Indications
Age ≥3 days of life, weight ≥700 g
Hemodynamically significant PDA (clinical and echocardiographic criteria)
Failure of, or contraindication to medical treatment
Stable for transport (neonatal team assessment)
Relative contraindications
Procedural weight <700 g
Minimal ductal diameter >4 mm
Minimal ductal length <3 mm
pre-existing LPA stenosis (LPA peak velocity >2 m/s)
pre-existing coarctation of the aorta (descending aorta peak velocity >2 m/s with a diastolic tail)
Intracardiac thrombus that may interfere with the procedure
Absolute contraindications
Active infection
Pulmonary hypertension with right-to-left ductal shunt
Ductal-dependent congenital heart disease

PA = left pulmonary artery; PDA = patent ductus arteriosus.

## Case planning

**Figure 2:**
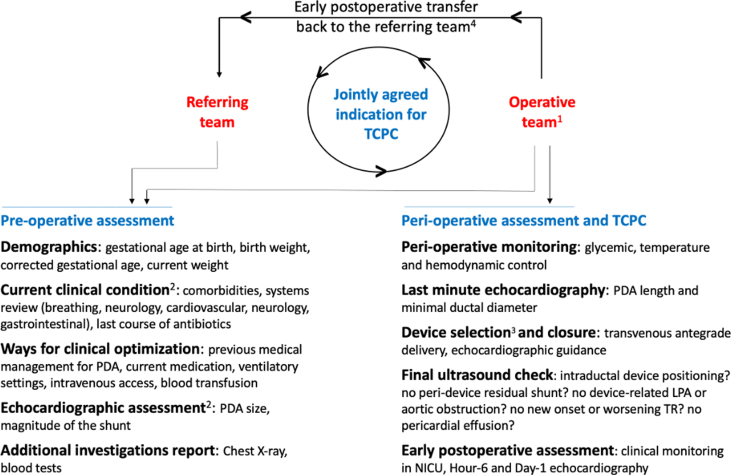
**Patient Clinical Pathway for Case Planning** ^1^Multidisciplinary: pediatric cardiology, neonatal intensive care, anesthesia; ^2^refer to appropriate PDA staging system; ^3^refer to the manufacturer’s instructions for use; ^4^as soon as possible based on clinical status, usually at postoperative day 1. LPA = left pulmonary artery; PDA = patent ductus arteriosus; TR = tricuspid regurgitation; TCPC = transcatheter PDA closure.

The indication for the device closure of a PDA in an ELBW infant is agreed upon by consensus among the treating neonatologists and pediatric interventional cardiologists. The decision is informed by a comprehensive pre-operative assessment encompassing demographic parameters, careful review of the current clinical situation, strategies for potential clinical optimization, and detailed echocardiographic evaluation (Figure 2). Once the indication of PDA closure is jointly agreed, the patient is transferred to the neonatal intensive care unit (NICU) at the institution where the procedure will be performed 1 day prior to the procedure. A comprehensive clinical assessment and echocardiogram is conducted to assess for any contraindications and parental informed consent is obtained. Hemoglobin and platelets are optimized as required. Intravascular access is secured before transfer to the catheterization laboratory to reduce the time spent outside the NICU. Two intravenous lines are established, for procedural sedation and resuscitation if required. If the patient is not already on mechanical ventilation, intubation is performed in the NICU. Mechanical ventilation is optimized to allow as much as possible respiratory stability during the procedure. Some institutions attempt a trial of conventional ventilation the day before the procedure in neonates dependent on high-frequency ventilation.^10^ The infant is transferred to the catheterization laboratory by the treating NICU team with the neonatal ventilator, avoiding disconnection from the ventilator for procedural purpose.^11^^,^^12^ Transcatheter PDA closure is routinely performed in the catheterization laboratory worldwide, although successful bedside interventions have been reported with the use of a portable C-arm fluoroscopy system or solely by echocardiographic guidance (ie, without fluoroscopy).^13^

## Echocardiographic guidance

Comprehensive PDA ultrasound assessment and careful transthoracic echocardiographic (TTE) guidance during the procedure is fundamental to procedural success and allows minimizing the use of both X-ray and intravenous contrast in this very fragile population.

The procedure can be entirely directed by ultrasound, by providing real-time imaging of the guidewire and catheter advancement within the heart.

**Figure 3:**
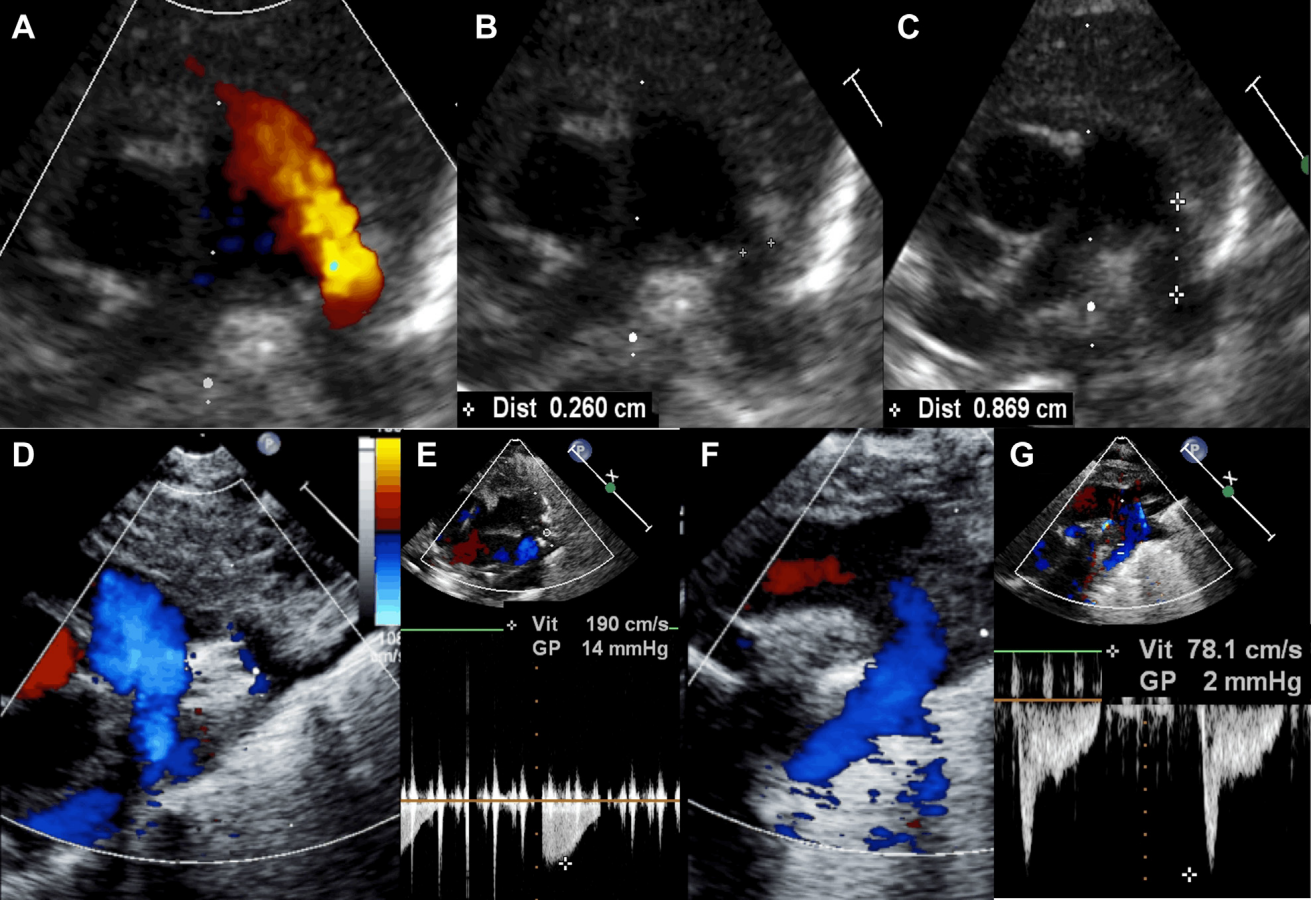
**Ultrasound Guidance of Transcatheter PDA Closure in ELBW Infants** (A to C) Last-Minute pre-procedural echocardiography. In the catheterization laboratory, before starting the case, a last-Minute echocardiography defines the best ultrasound window and carefully measures the ductal length and the minimal ductal diameter. Here is a large, left-to-right shunting, patent ductus arteriosus in an ex-24 weeker premature infant, with a procedural weight of 740 g (A). The minimal ductal diameter is measured at 2.6 mm (B), with a ductal length of 8.7 mm (C). (D to G) Real-time echocardiographic guidance. Ultrasound check of a well-positioned 4/2 Amplatzer Piccolo Occluder device, with no residual shunt (D and F) and no device-induced left pulmonary artery obstruction (D and E) (Vmax 1.9 m/s) or descending aorta obstruction (F and G) (Vmax 0.8 m/s). Note that the procedure is performed in an infant on high-frequency jet ventilation.

Prior to commencing the procedure, a final echocardiogram assists in determining the optimal echocardiographic window, which can be difficult particularly in preterm infants requiring high frequency ventilation or those with severe bronchopulmonary dysplasia. Meticulous measurement of the PDA minimal ductal diameter and length is essential to selecting the appropriate device (Figure 3).

Echocardiographic guidance for device positioning relies on a high parasternal view. A satisfactory result is defined by an intraductal device position, no residual peri-device shunt, no device-related left pulmonary artery (LPA) obstruction (LPA peak velocity <2.5 m/s), and no device-related descending aortic obstruction (descending aorta, DAo peak velocity <2.5 m/s) (Figure 3).^6^

Following device release, careful TTE assessment verifies procedural success and ensures the absence of new-onset or worsening tricuspid regurgitation or pericardial effusion.

## Anesthesia

**Figure 4:**
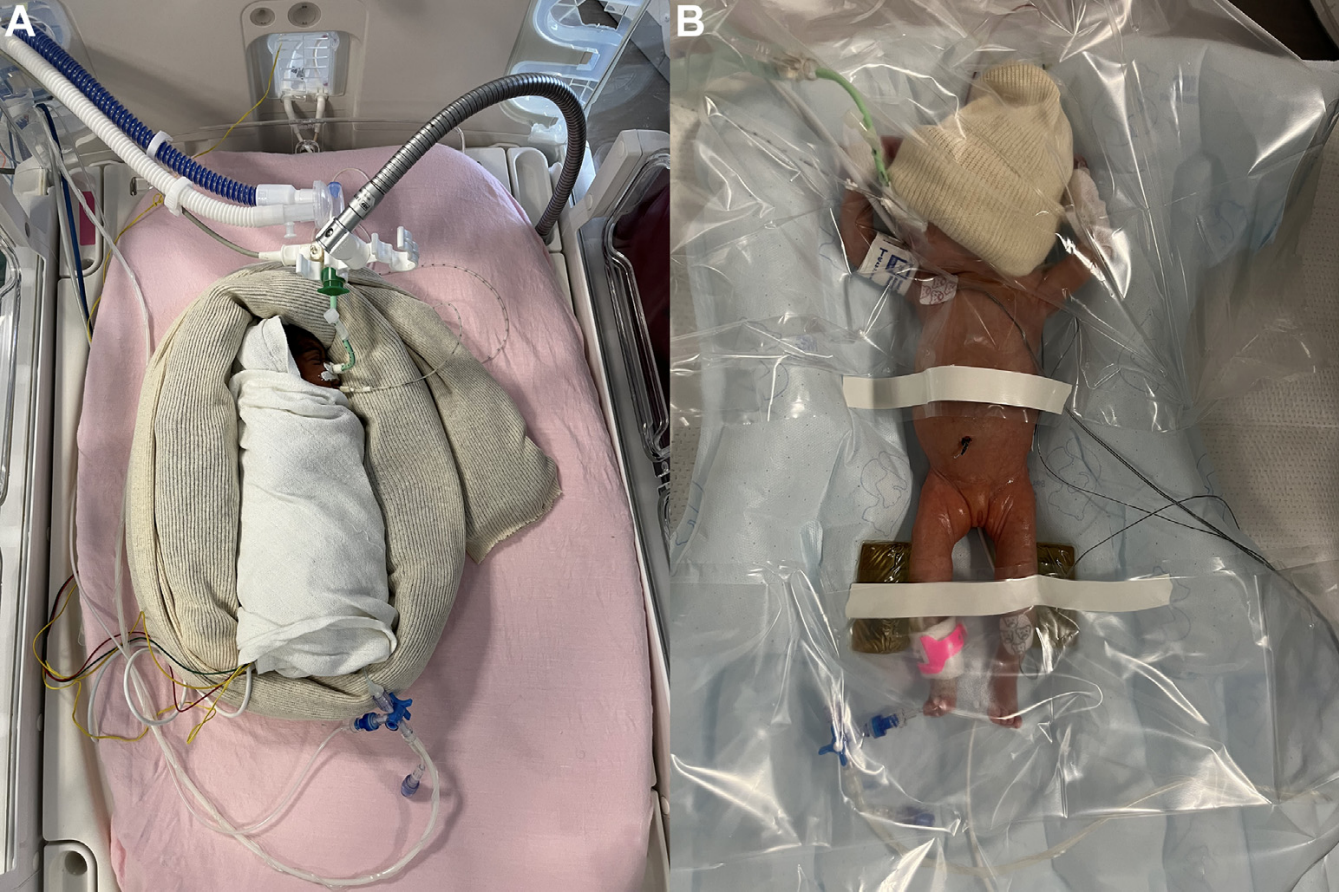
Prevention of Hypothermia (A) During infant’s transport from NICU to the catheterization laboratory and round trip; (B) on table, during the procedure.

Although small, an ELBW infant requires substantial space in the catheterization laboratory to accommodate the ventilator and other neonatal equipment. The anesthetic management is based on four main objectives: 1) effective sedation and analgesia, which is commenced before patient transportation to the procedure room and adjusted accordingly during the procedure; 2) careful continuous monitoring of near-infrared spectrometry, pre- and post-ductal oxygen saturations, electrocardiogram, noninvasive blood pressure, esophageal temperature, and end-tidal carbon dioxide; 3) safe patient transport from the NICU to the catheterization laboratory, keeping in mind that even short distances within the hospital pose risks such as hypothermia, loss of vascular access, disruption of medication infusions, or accidental extubation for ELBW infants.^11^^,^^12^ It is thus paramount to be adequately prepared as a team, with an emergency kit and back-up equipment; 4) prevention of hypothermia, of which ELBW infants are vulnerable due to their high surface area to body weight ratio as well as the lack of an insulating fat layer. Hypothermia may expose to hypoglycemia, metabolic acidosis, pulmonary hypertension, and hypoperfusion of vital organs. Mitigating hypothermia is based on well-defined good-practice interventions,^14^ that is, pre-emptive heating, continuous central temperature monitoring by an esophageal probe, and appropriate measures to avoid cooling such as warm blankets and covering the patient (Figure 4). The procedure should only be commenced once the patient is stable and all equipment is secured.

## Device selection

PDA coil occlusion was successfully reported in 2007 in a historical series of 10 preterm infants with a procedural weight between 1,600 and 2,650 g;^15^ it has gradually been superseded by a few devices which have been successfully used in closing preterm PDAs, including the AVP-II,^16^, ^17^, ^18^ the AVP IV (Abbott),^19^ the micro vascular plug (Medtronic),^20^ the Micro Plug Set (Micro Plug, KA Medical),^21^^,^^22^ and the Amplatzer Duct Occluder II Additional Sizes renamed the APO after the pivotal US clinical trial.^23^ Technical characteristics and potential drawbacks of the available devices are summarized in Table 3. The length of the AVP-II, AVP-IV, and MVP may be a limiting factor in increasing the risk of DAo and/or LPA obstruction. Moreover, the MVP, comprised of a nitinol framework partially covered by a polytetrafluoroethylene membrane at the proximal portion, exhibits low radio-opacity making visualization difficult.^24^ The Micro Plug is a new microcatheter-delivered device with promising safety and efficacy reported in a single-center series of 25 patients,^22^ although this device is not yet commercially available in the European market. The APO is currently the only dedicated device available for this procedure; it has a particular design for both the fetal duct morphology and the nonelongated tubular ductus with a narrowing on the pulmonary side (Hockey stick morphology). It has been Conformité Européenne–marked and FDA-approved for use in ≥700 g and day ≥3 of life premature infants with a ductal length ≥3 mm and a minimal ductal diameter ≤4 mm. It is a self-expandable, nitinol mesh device with a central cylindrical waist and low-profile retention discs on both ends that are marginally larger than the waist, resulting in a nearly isodiametric device. The device comes pre-loaded on a delivery cable and can be delivered through a 4-F Amplatzer TorqVue LP catheter (Abbott Structural Heart). The APO is available in nine sizes comprised of three waist diameters (3, 4, and 5 mm) and three lengths (2, 4, and 6 mm).

**Table 3:** Used Devices for PDA Closure in ELBW Infants

Device	Device Waist Diameters	Device Length	Delivery Sheath	Pros	Cons
Approved device					
APO (Abbott)	3, 4, 5 mm	2, 4, 6 mm	4-F	The only CE-marked and FDA-approved device	None
Off-label used devices					
AVP-II (Abbott)	6, 8 mm^a^	6 mm	5-F	Suitable for tubular PDAs with a diameter larger than 4 mm	Increased risk of DAo and/or LPA obstruction because of a rather long device
AVP-IV (Abbott)	6, 7, 8 mm^a^	11, 12.5, 13.5 mm, respectively	4-F	Suitable for tubular PDAs with a diameter larger than 4 mm	Increased risk of DAo and/or LPA obstruction because of a rather long device
MVP (Medtronic)	5.3 (3Q), 6.5 (5Q), 9.2 (7Q) mm	12 (3Q, 5Q), 16 (7Q) mm	4-F	Microcatheter delivered device	Increased risk of DAo and/or LPA obstruction because of a rather long device Lack of radio-opacity
Micro Plug Set (KA Medical)	3, 4, 5, 6 mm	2.5 mm	4-F	Microcatheter delivered device	Limited experience so far Not currently available in the European market

APO = Amplatzer Piccolo Occluder; AVP-II = Amplatzer Vascular Plug-II; AVP-IV = Amplatzer Vascular Plug-IV; DAo = descending aorta; MVP = micro vascular plug; LPA = left pulmonary artery; PDA = patent ductus arteriosus.

a Additional sizes exist but are not suitable for PDA closure in ELBW infants.

## Procedure description

Transcatheter PDA closure is usually performed in the catheterization laboratory under general anesthesia, with the patient connected to their own ventilator. The procedure is performed with both biplane fluoroscopy and TTE guidance. A 4-F sheath is inserted in the femoral vein, under ultrasound guidance to reduce the risk of access complications, particularly inadvertent puncture of the femoral artery. Prophylactic antibiotics are administered. There is no consensus on prophylactic heparin administration: some operators give 50 to 100 U/kg of unfractionated heparin as a bolus once access has been achieved,^23^^,^^25^ whereas others only use heparinized saline to flush the catheters before insertion with no direct administration of heparin into the patient.^24^^,^^25^

Procedural steps are standardized and streamlined to minimize operative time as much as possible. Although there are significant variations in practice across centers, device closure of PDA in ELBW infants differs from that in infants weighing over 2 kg and older patients in the following aspects which are standardized worldwide: 1) the device is positioned and delivered from the venous side, using materials of the smallest size feasible; 2) no arterial access is obtained; 3) complete hemodynamic work-up is not routinely performed to shorten the procedure time and minimize catheter manipulation in these vulnerable patients; 4) procedural guidance relies heavily on TTE to minimize radiation exposure, given the increased radiosensitivity of young infants, higher heart rates, smaller cardiovascular structures, and smaller body size, all of which remain specific challenges. Despite adhering to the ALARA (As Low As Reasonably Achievable) concept, there is still a risk of relatively high radiation doses to the patient and the potential to develop radiation-related sequelae, including increased standardized incidence ratios for all-cancer, leukemia, lymphoma, and solid cancers compared with the general population;^26^ 5) a limited use of contrast through the catheter placed in the DAo—and in some groups, no use of contrast. This provides accurate PDA delineation and aids in appropriate device selection as well as device positioning based on contrast landmarks in addition to the TTE assessment.

A last-minute TTE assessment is made on the table, to define the best echocardiographic window and to measure the minimal ductal diameter and the ductal length.^27^ A catheter led by a soft 0.014-inch wire is advanced from the femoral vein through the right heart and positioned across the PDA into the DAo. It is worth noting that a transjugular venous approach for PDA closure with the APO has been occasionally reported in VLBW infants with either interrupted or obstructed inferior vena cava.^28^ Various techniques are used worldwide to position the 4-F TorqVue LP delivery catheter across the PDA in the DAo (Supplemental Appendix).

As per the manufacturer’s instructions for use, the device diameter is selected to be 1 mm larger than the PDA diameter, and the device length corresponds to the PDA length (2 mm APO length if PDA length <12 mm, 4 mm APO length if PDA length ≥12 mm), to avoid protrusion of the device in the DAo and/or in the LPA.^29^ As a rule, the device is placed entirely intraductal in infants weighing <2,000 g, avoiding any uncovered PDA segment at the pulmonary end (Central Illustration).^30^ Successful positioning is defined by complete occlusion of the duct with no peri-device residual shunt and the absence of aortic or LPA obstruction by echocardiography using 2D, color and spectral Doppler. The device may need to be reloaded and repositioned if TTE demonstrates color flow aliasing on adjacent vessels (ie, DAo and LPA), a peak velocity >2.5 m/s on pulse wave Doppler, or persistent antegrade diastolic flow.^6^ A smaller device may be attempted if felt reasonable by the operator. Following the release of the device, careful echocardiographic assessment is repeated, paying particular attention to the tricuspid valve function and to the pericardial space. The patient is then transferred back to the NICU for close clinical monitoring and follow-up echocardiography within the first post-procedural 24 hours.

## Procedural early outcomes

Transcatheter PDA occlusion in premature infants weighing ≤2,000 g using the APO device has been reported to be highly effective with a success rate exceeding 98% in recent large-scale series, including the multicenter American prospective trial (N = 100, procedural weight: 1,250 ± 350 g, success rate: 99%),^23^ the multicenter French cohort study (N = 102, procedural weight: 1,543 ± 698 g, success rate: 99%),^24^ and a multicenter international study (N = 68, procedural weight: 1,200 ± 370 g, success rate: 98%).^25^ ELBW infants did not experience post-PDA ligation syndrome after transcatheter PDA occlusion.^31^ Additionally, they demonstrated faster respiratory improvement and shorter mechanical ventilation,^18^^,^^26^^,^^32^ as well as less major complications and less overall mortality^31^ when compared to matched cases with surgical PDA ligation by posterior thoracotomy. Preterm infants were discharged home earlier than matched surgical cases when device closure is performed before the fourth week of age.^26^ It has however been suggested that the surgical minimally-invasive approach by anterior mini-thoracotomy may provide equivalent safety and efficacy than device PDA closure in ELBW and VLBW infants.^33^

**Figure 5:**
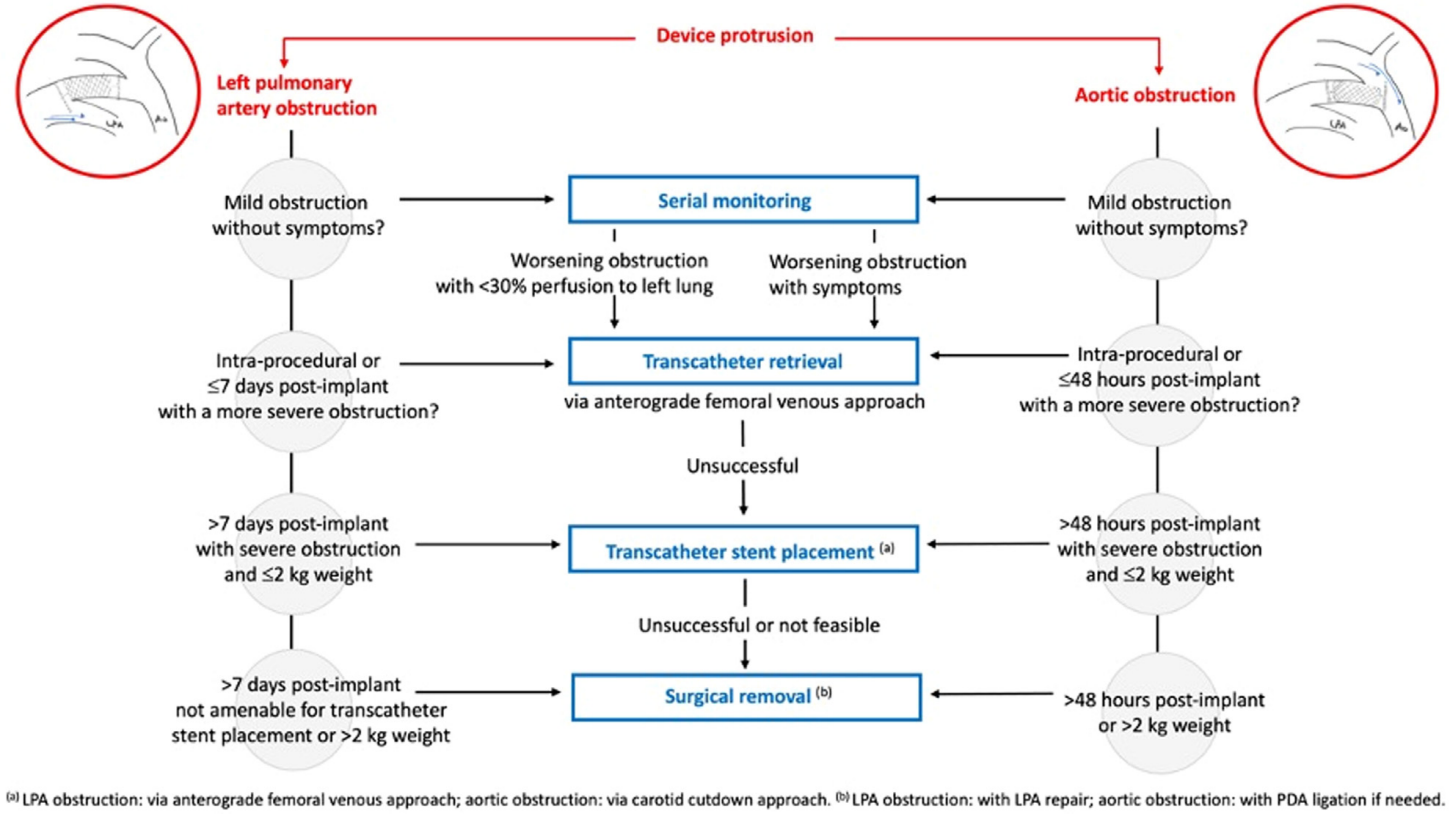
**Management Algorithm for Device-Induced Left Pulmonary Artery or Aortic Obstruction** Adapted from Consensus guidelines, Sathanandam et al.^6^ LPA = left pulmonary artery; PDA = patent ductus arteriosus.

On review of published studies with >20 cases of transcatheter PDA closure in ELBW infants, to avoid considering complications related to the learning curve, procedure-related mortality is very low (Table 4) (1/347 patients, 0.3% mortality rate) and possibly associated with the learning curve in historical series. In 2017, Morville et al. reported one procedural death out of 32 patients, due to cardiac perforation in a 680-g infant.^34^ Since this case, there have been no procedural deaths reported in the 318 successful APO implantations in <2 kg infants.^23^, ^24^, ^25^^,^^32^^,^^35^^,^^36^ Procedure-related MAE consist of device embolization (2.3%, percutaneous device retrieval in all cases), device-induced aortic obstruction (1.4%), device-induced LPA obstruction (2.0%), and cardiovascular injury (0.9%) (Table 4).^29^ It is important to note that with increasing experience, the incidence of MAE notably declines, decreasing from a 10 to 15% rate of MAE in series with <30 patients^32^^,^^34^, ^35^, ^36^ to a 4 to 5% rate of MAE in series with >60 patients (Table 4).^23^, ^24^, ^25^ The incidence of new onset or worsening tricuspid valve regurgitation in <2 kg infants was 5% in the premarket trial ^23^ and 4.1% in the French multicenter study.^24^ The most common cause of tricuspid regurgitation is injury to the chordae of the septal leaflet of the tricuspid valve, which may be damaged during the catheter course across the valve. The incidence, mechanisms, and guidelines for the prevention and management of major procedural complications have been summarized in a recent expert consensus statement.^6^ Device-related complications and algorithms for the management of device-induced left pulmonary obstruction or aortic obstruction are summarized in Table 5 and Figure 5, respectively.

**Table 4:** Procedural Safety of Transcatheter Patent Ductus Arteriosus in <2,000 g Premature Infants Using the Amplatzer Piccolo Occluder

First Author, Year	N	Country	Procedural Weight (kg)	Success Rate (%)	Major AE (%)	Device Embolization	Aortic Obstruction	LPA Obstruction	Cardiovascular Injury	Procedural Mortality
Morville, 2017^34^	32	France	1.37 (0.68-2.48)	31/32 (97%)	9.4	0 (0.0%)	0 (0.0%)	1 (3.1%)	1 (3.1%)^a^	1 (3.1%)
Rodriguez, 2018^32^	27	Spain	1.26 (1.00-1.98)	27/27 (100%)	11.1	2 (7.4%)	0 (0.0%)	0 (0.0%)	1 (3.7%)^a^	0 (0.0%)
Pamukcu, 2018^35^	26	Turkey	1.39 (0.75-2.00)	22/26 (85%)	15.4	2 (7.7%)	1 (3.8%)	0 (0.0%)	1 (3.8%)^a^	0 (0.0%)
Regan, 2020^25^	64	UK, France	1.20 (1.02-1.70)	63/64 (98%)	4.7	2 (3.1%)	1 (1.6%)	0 (0.0%)	0 (0.0%)	0 (0.0%)
Milani, 2020^24^	73	France	<2,000 g	73/73 (100%)	4.1	0 (0.0%)	0 (0.0)	3 (4.1)	0 (0.0%)	0 (0.0%)
Sathanandam, 2020^23^	100	USA	1.25 (0.7-2.00)	99/100 (99%)	4.0	2 (2.0%)	2 (2.0%)	0 (0.0%)	0 (0.0%)	0 (0.0%)
Wang, 2021^36^	25	Taiwan	1.21 (0.48-1.98)	25/25 (100%)	16.0	0 (0.0%)	1 (4.0%)	3 (12.0%)	0 (0.0%)	0 (0.0%)
**All**	**350**		<2,000 g	**340/347 (98%)**		**8/347 (2.3%)**	**5/347 (1.4%)**	**7/347 (2.0%)**	**3/347 (0.9%)**	**1/347 (0.3%)**

Studies were selected if >20 cases had antegrade delivery of an Amplatzer Piccolo Occluder. The Amplatzer Piccolo Occluder is the only device reported in Table 1, because this is currently the only minimally invasive PDA closure device that is FDA-approved and CE-marked for premature infants. Adapted from Baruteau et al.^29^

AE = clinically relevant, procedure-related adverse event.

a Cardiac tamponade.

**Table 5:** Device-Related Complications

Complications	How to Avoid	How to Manage
Device embolization	• Accurate assessment of the PDA dimensions (last-minute TTE (prior to instrumentation) ± angiography. • Appropriate device selection and sizing. • Appropriate device positioning (intraductally in <2 kg infants). • Comprehensive TTE before device release, ruling out any peri-device residual shunt.	• Administer heparin for ACT >200 s. • Get ready for blood transfusion in the event of excessive blood loss due to catheter exchange. • Select a 4F diagnostic catheter for snaring the device, along with a suitable retrieval sheath placed in the MPA (otherwise in the RV or RA). • consider gently unguarded device retrieval if the sheath cannot be safely advanced into the MPA. • In case of aortic embolization, consider device retrieval via the PDA, via a carotid approach or surgically. • Onsite surgeon. Requested materials Retrieval sheath: 4-F or 5-F Cook Flexor Ansel guiding sheath with check-flo hemostatis valve (the 4-F TorqVue LP catheter cannot be used as a retrieval sheath). • Diagnostic catheter for accessing RPA: 4-F Judkins Right 2.0 or 2.5. • Diagnostic catheter for accessing LPA: 3.3-F Mongoose JB1 or JR2. • Snares: 3.2-F Merit Ensnare or 5 mm Amplatz Gooseneck snare.
Device protrusion and aortic and LPA obstruction	• Double check any pre-existing LPA stenosis or aortic coarctation before PDA instrumentation. • Appropriate device length selection (2 mm length in infants <1 kg, 4 mm length only if ductal length >12 mm). • Appropriate device positioning (intraductally in <2 kg infants). • Use the esophageal temperature probe as a fluoroscopic landmark of the aortic isthmus in infants <2 kg. • Consider deploying the aortic disc within the PDA rather than in the DAo to avoid protrusion of its superior edge in the aortic lumen.	• Refer to management algorithm (Figure 4)

Adapted from Sathanandam et al.^6^

ACT = activated coagulation time; DAo = descending aorta; LPA = left pulmonary artery; MPA = main pulmonary artery; PDA = patent ductus arteriosus; RA = right atrium; RPA = right pulmonary artery; RV = right ventricle; TTE = transthoracic echocardiography.

## Mid- to long-term outcomes

Regular TTE follow-up is paramount to exclude any delayed device protrusion and to monitor LPA and descending aortic flow velocities. In the absence of significant, progressive device-induced vascular obstruction in the early post-procedure period, mild increase in LPA and DAo flow velocities (Vmax <2.5 m/s) tend to improve spontaneously and normalize in long-term follow-up.^37^ Delayed occurrence of device-induced, clinically significant aortic coarctation has been reported in ELBW, underlining the importance of closely monitoring of the DAo flow velocity at least up to the end of the first month after device implantation.^30^ Longer term follow-up data is provided by the U.S. multicenter prospective study (NCT03055858), in which overall patient survival at 3 years was 92.9% in the 100 patients who had a procedural weight <2,000 g.^38^ Of the 7 patients weighing <2,000 g who died following device implantation, no deaths were attributed to the device or procedure following an independent review by the clinical events classification committee. Beyond 6 months, there were no additional device or procedure-related complications reported. Importantly, worsening of postprocedural tricuspid regurgitation was observed in 5 ELBW (5/100, 5%) with 2 cases classified as severe; however, no intervention was required.^38^

## Future directions

Transcatheter PDA closure is likely to become available to a larger number of preterm ELBW infants. As catheters and delivery systems become increasingly scaled-down in size, it is anticipated that further improvements, including additional FDA-approved devices and fluoroscopy-free percutaneous approaches, will continue to improve the safety and efficacy of transcatheter PDA closure in ELBW infants. Developing a fluoroscopy-free intervention at the patient’s bedspace, without angiography and with echocardiographic guidance only would be a major step forward in the management of these extremely preterm infants, in whom the benefit-risk balance would be in favor of avoiding the potential risks associated with both radiation and contrast use. In 2011, Bentham et al^39^ reported successful fluoroscopy-free device PDA closure on 3 premature infants, although the approach for device deployment was retrograde arterial in these cases. Bedside transcatheter PDA closure solely guided by echocardiography within the neonatal intensive care unit environment has also been successfully reported in a 790 g ELBW infant^40^ and in a consecutive series of 11 premature infants between 800 and 1,600 g.^13^ If demonstrated to be safe and effective in larger patient cohorts, this new technique, which eliminates both radiation and the need to transport these fragile patients to the catheterization laboratory, could emerge as the foundation for a new gold standard for PDA closure in preterm infants.

## Conclusions

Transcatheter PDA closure in unselected premature ELBW is increasingly recognized as an equally effective but safer alternative to surgical ligation in experienced centers. Although follow-up studies have reported favorable short- and medium-term outcomes, these findings need further validation in prospective trials comparing outcomes of this technique to current treatment strategies, including medical treatment. Moreover, data regarding the impact of this new nonpharmacological alternative on the neurodevelopmental outcomes and long-term respiratory status are currently lacking. There is a critical need for multicenter registries to report both early- and long-term results on a large scale and to further define the optimal timing for this procedure.

## Funding support and author disclosures

Dr Alban-Elouen Baruteau is supported by the French Government as part of the “Investments of the Future” program managed by the National Research Agency (grant reference ANR-16-IDEX-0007); and is a consultant and proctor for Abbott. All other authors have reported that they have no relationships relevant to the contents of this paper to disclose.
