# Supplementary material for: Device Closure of Hemodynamically Significant Patent Ductus Arteriosus in Premature Infants
Source: JACC Adv. 2024 Aug 24;3(10):101211. doi: 10.1016/j.jacadv.2024.101211 (PMC11388744; doi:10.1016/j.jacadv.2024.101211)
Supplement: Supplemental Data [file mmc1.docx]

**SUPPLEMENTAL APPENDIX**

**Procedure Description.** Some interventional teams use a 3.3 French Mongoose catheter (PediaVascular, Chagrin Falls, Ohio, USA) or 3.0 French multipurpose BALT catheter (Montmorency, France) over a 0.018-inch hydrophilic wire (Terumo) or a 0.014-inch soft coronary wire. This allows advancement of the 4-French sheath in the femoral vein, through the inferior vena cava towards the right atrium, right ventricle, across the PDA and positioning the catheter in the descending aorta. Sathanandam et al. have reported a similar technique using a 4 French-angled glide catheter (Terumo, Japan) and a 0.035-inch Wholey wire (Medtronic, Minneapolis, MN, USA) to cross the PDA anterograde into the descending aorta (1). Other teams have used a 4 French Swan-Ganz catheter (2).

Sometimes, the 4-French TorqVue LP delivery catheter can be directly advanced over a 2.7-French Progreat microcatheter and a coronary wire in a telescopic fashion (2). Alternatively, a 2.7-French Progreat microcatheter can be modified by cutting and removing the proximal hub. It can then be inserted in a 4French Judkins right coronary catheter 2.0, over a 0.014 inch Hi-Torque Pilot guidewire introduced through the microcatheter. The ensemble can be advanced into the right atrium and the microcatheter is used to cross the tricuspid valve. Once the microcatheter is positioned within the right ventricle, the 0.014-inch guidewire is advanced to the pulmonary artery and across the PDA and the microcatheter can be advanced to the descending aorta. The right coronary catheter, which always remains in the right atrium, is removed and the 4-French TorqVue LP delivery system is advanced over the microcatheter (3). If a 3 French catheter is used to cross the PDA, a 0.021 inch Fixed-Core Guide Wire (Cook Medical, USA) can be placed in the descending aorta and the 3-French catheter is directly exchanged with the 4-French TorqVue LP delivery sheath. The distal tip of the TorqVue sheath is placed in the descending aorta slightly distal to the PDA.

**References**

1. Herron C, Forbes TJ, Kobayashi D. Transjugular venous approach for Piccolo patent ductus arteriosus closure in a 1.4 kg infant. Cardiol Young. 2022;32:111-112.

2. Roberts P, Adwani S, Archer N, Wilson N. Catheter closure of the arterial duct in preterm infants. Arch Dis Child Fetal Neonatal Ed. 2007;92:F248-50.

3. Philip R, Tailor N, Johnson JN, et al. Single-Center Experience of 100 Consecutive Percutaneous Patent Ductus Arteriosus Closures in Infants 1000 Grams. Circ Cardiovasc Interv. 2021;14:e010600.
